# Spatial-Temporal Event Analysis as a Prospective Approach for Signalling Emerging Food Fraud-Related Anomalies in Supply Chains

**DOI:** 10.3390/foods12010061

**Published:** 2022-12-22

**Authors:** Ana M. Jiménez-Carvelo, Pengfei Li, Sara W. Erasmus, Hui Wang, Saskia M. van Ruth

**Affiliations:** 1 Food Quality and Design, Wageningen University and Research, P.O. Box 17, 6700 AA Wageningen, The Netherlands; 2 Department of Analytical Chemistry, Faculty of Sciences, University of Granada, C/Fuentenueva, s/n, E-18071 Granada, Spain; 3 School of Electronics, Electrical Engineering and Computer Science, Queen’s University Belfast, Belfast BT9 5BN, UK; 4 Institute for Global Food Security, School of Biological Sciences, Queen’s University, 19 Chlorine Gardens, Belfast BT9 5DL, UK; 5 UCD School of Agriculture and Food Science, University College Dublin, 4 Dublin, Ireland

**Keywords:** food crime, food fraud, smart tags, spatial-temporal data, supply chain, traceability

## Abstract

One of the pillars on which food traceability systems are based is the unique identification and recording of products and batches along the supply chain. Patterns of these identification codes in time and place may provide useful information on emerging food frauds. The scanning of codes on food packaging by users results in interesting spatial-temporal datasets. The analysis of these data using artificial intelligence could advance current food fraud detection approaches. Spatial-temporal patterns of the scanned codes could reveal emerging anomalies in supply chains as a result of food fraud in the chain. These patterns have not been studied yet, but in other areas, such as biology, medicine, credit card fraud, etc., parallel approaches have been developed, and are discussed in this paper. This paper projects these approaches for transfer and implementation in food supply chains in view of future applications for early warning of emerging food frauds.

## 1. Introduction

Food fraud is an emerging international issue that is often defined as the deliberate and intentional adulteration, substitution, dilution, simulation, tampering, counterfeiting or imitation of food, food ingredients or food packaging; or false or misleading label claims made about a product for economic gain [1,2]. Hence, food fraud is economically motivated but does not necessarily involve a health hazard [3,4]. However, in some cases consumers do pay for these practices with their health, and in the worst cases, even with their lives [5,6]. Food fraud is most likely to occur when a motivated offender and opportunity arises in the absence of control measures [1,7]. Food fraud can be divided into various kinds, which are outlined below [3,8,9,10]:

Adulteration. The food is added with one or some substances in a premeditated and intentional manner to cover up some defects in nutrition, weight, color, etc.

Substitution The high-value ingredient or part of the food is replaced by that of lower value to increase its perceived value or reduce the cost of its production.

Simulation. It consists of forming, ex novo, a food that may appear to be the original.

Counterfeiting. The faking of a higher quality product with one of lesser quality to obtain an illicit economic benefit.

Theft. Dishonestly obtaining food, drink, or feed products to profit from their use or sale.

Misrepresentation/mislabeling. The food has not been properly labelled and does not correctly cite the list of ingredients it contains or involves marketing which may wrongly portray its quality, safety, origin, or freshness.

Product overrun. The product did not pass the standards either with factory defects or was slightly damaged, or there is a product made in excess of production agreements.

Packaging recycling. The original product is replaced with a lower quality product. This type of fraud is common in beverages, as with wine, for example.

Diversion. The sale or distribution of legitimate products outside of the intended markets.

Waste diversion. This involves the illegal diverting of food or drink meant for disposal back into the supply chain. In this case, the label of the product can be counterfeited, changing the expiration date of the food. 

Document fraud. Making, using, or possessing dishonest documents with the objective of selling or marketing a fraudulent or substandard food.

In this context, the involvement of all participants (primary/secondary/tertiary processors; wholesalers; brokers etc.) in the supply chain is crucial, since it involves all of the operations that are indispensable for a product to reach the final customer in optimal conditions [11]. 

However, although there are three main segments in the supply chain, each involves several stages and nodes that make the process complex and, therefore, transparency at every stage is crucial to avoid any kind of food fraud. In other words, the supply chain is the complete life cycle of a food product, involving each step from raw material to final sale; therefore, it requires coordination among all the nodes/steps in the chain.

In this regard, the Global Food Safety Initiative (GFSI) is an important global collaborative platform that is industry-driven and aims to provide leadership and guidance on food safety management systems, such as the International Featured Standards (IFS), Food Safety System Certification (FSSC 22000), and Brand Reputation through Compliance of Global Standards (BRC standards) [12,13,14]. In 2014, GFSI [15] recognised the importance of the fight against food fraud by recommending two fundamental steps to be taken to prevent food fraud. The first was to carry out a ‘food fraud vulnerability assessment’ in which information from the supply chain (raw materials, packaging, etc.) was collected. The SSAFE food fraud vulnerability self-assessment tool was developed by SSAFE in collaboration with Wageningen University, the Free University of Amsterdam, PwC, and many stakeholders, and was provided free of charge to the food industry (www.ssafe-food.org, accessed on 16 October 2022). In the assessment, various technical and managerial control measures are considered. Technical control measures include fraud monitoring systems based on analytical testing, mass balance information systems, as well as track and trace systems. 

In these traceability systems, interesting data of product identifiers in time and place are collected. These spatial-temporal data may be an interesting source for the early warning of emerging frauds, which is explored in the current paper. This commentary paper is divided into four sections. Following this introduction, the second section presents the common procedures of food traceability systems, the third section discusses spatial-temporal analysis and its potential application in the food industry, and the last section is devoted to future prospects, which includes a discussion of the challenges and limitations of this approach. 

## 2. Current Track and Trace Practices in the Food Industry

Food traceability allows for the tracing of all of the steps that a food has followed from its origin, through its transformation process, and ending up in the hands of consumers. Currently, within food traceability, it is possible to designate two approaches to ensure the authenticity of a food: (i) the intrinsic approach, based on markers in the product, which may change throughout the chain, with tracers added or naturally present, etc., and (ii) the extrinsic or digital approach based on controlling product labelling using barcodes, smart packaging, etc., since most goods are identified by labelling and contain a basic barcode that must comply with international standards for food traceability [16].

Regarding digital information systems, there are many information and communication technology-based systems applied in the industry. Examples would be the use of radio frequency tags (RFID) or near-field communication (NFC). Similarly, the use of labels printed with bi-directional quick response codes (QR codes) provides consumers with information on the origin of the product they are buying, giving them added value. This provides an excellent link between the physical world and the internet, since consumers could easily access the full history of a product by simply using their mobile devices. Therefore, the use of QR codes is having an increasing impact on how brands present and sell their products that they distribute at retail.

In fact, it would be possible to achieve the forward and backward traceability of primary, secondary and tertiary industrial packaging of food after reading these codes, as it would be possible to obtain real-time, certified, and geo-localised information on the situation of the packaging at any given moment. The task of data and information assignment to the products and the task of reading or accessing the information could be automated by these tags. In this regard, there is one Spanish company which focuses its business activity on the traceability of packaging by means of GPS coordinates (position coordinates) obtained when scanning the QR codes on the packaging (https://qrtracing.com/ accessed on 16 October 2022).

The spatial-temporal data generated in the current traceability systems may potentially also serve as an early warning source to flag suspected anomalies in the chain. 

## 3. Spatial-Temporal Data Analysis

In recent years, technological advances derived from Artificial Intelligence (AI), nanotechnology, robotics, the Internet of Things (IoT), i.e., the so-called fourth industrial revolution (IR 4.0), are accentuating the possibilities of unprecedented processing, storage, and access to knowledge [17,18]. Big Data and technologies are ever-present. Indeed, in the last few years we have been living in the transition from the IoT to the so-called Internet of Everything, applying the most innovative technologies that facilitate the digitisation of processes and providing an innovative solution for the control and traceability of assets in the supply chain. IoT is a collective network of connected devices and the technology that facilitates communication between devices and the cloud, as well as between the devices themselves. Data on food product identifiers can be collected along the chain by using these technologies. AI can be used to make sense of the data. AI calculations can fuse the data of different sources along the chain and provide input for decision support tools to enhance food quality and safety. As a result of all of this, logistics and distribution companies are investing in technologies that enable real-time tracking of the product along the entire supply chain, from the moment it leaves the supplier to the final consumer [19,20]. In this sense, thanks to the advance of new technologies, it is possible to record the movement of goods after scanning a product’s identification code, giving rise to real-time reading, leading to "spatial-temporal" databases (a combination of spatial and temporal data). Blockchain technology (BCT) allows the unmodifiable storage of the traceability along the chain [21,22]. BCT is a chronological data structure in which transactions are grouped into groups or blocks which are then recorded identically in a computer network [23]. However, it is important to note that it is only a means to ensure that data cannot be modified after it has been entered into the system. It does not prevent the upload of incorrect or falsified information. Moreover, it has a high degree of complexity, a significant verification mechanism and cost of implementation, and the confidentiality and data protection are some of the issues that need to be overcome to optimally implement BCT in the food chain.

At this point, however, a very important aspect must be taken into account: the protection of the information collected. Data protection is focused on two important aspects: security (protects data) and privacy (protects identity). For this reason, the use of this type of data would imply implementing the necessary security measures to ensure that the privacy of the information and data transferred is maintained throughout the entire life cycle of the data.

Temporal data are observations that refer to different moments in time, while spatial data refer to different geographic locations. In other words, the main difference between the two types of data is that they refer to different dimensions. The temporal dimension is linear (past-present-future), while the spatial dimension is two-dimensional, i.e., the geographic data refer to locations that are related to each other in several directions (north, south, east, and west), which implies a greater complexity in the observations. Thus, spatial-temporal databases host data collected across both space and time that describe an event in a particular location and period of time. The information stored in spatial-temporal databases depends on certain characteristics, namely: (i) the spatial domain, (ii) the temporal domain, and (iii) movement and change. The latter characteristic refers, on the one hand, to movement due to alterations in position in space over time; and on the other hand, change deals with how the spatial object undergoes transformation in its extension.

Note that the study of spatial-temporal data is a tool that has been applied for years in different scientific fields (such as spatial data_ by using geographic information systems (GIS) for different purposes, one of the most recent of which is for the respiratory syndrome coronavirus 2 (SARS-CoV-2) pandemic [24,25]. However, its implementation as a tool to control the traceability of goods has not been fully exploited, since it requires the application of probabilistic and statistical models for the analysis of high-dimensional data that often exhibit complex correlation structures in time and/or space (globalisation). 

### 3.1. Examples of Spatial-Temporal Data Analysis Applications Outside the food Traceability Domain

The analysis of spatial-temporal data is commonly used with the aim of testing certain hypotheses, models, or to study the behaviour of events in different areas. The following involves examples of some areas in which this type of analysis is carried out.

Applications for spatial-temporal data analysis include the study of marine biology, in this case the spatial-temporal analysis of a shoal of fish which allowed the determination of the movement of the shoal, and to know in which season they migrate or even to determine whether fishing should be avoided [26]. In another area, such as medicine, the spatial-temporal analysis of a virus can be used to determine its level of evolution/mutation and/or the degree of particular spread of an epidemic, as for example in the current severe acute respiratory syndrome coronavirus 2 (SARS-CoV-2) pandemic [27,28]. In the cosmetics sector, Benatia et al. [29] described an approach to follow products along the supply chain. They proposed the use of spatial-temporal data and the employment of an enumeration tree algorithm that provides products with genuine trajectories based on simulation data. Each transaction was represented by identification (ID), action, location, timestamp, and duration. The goal was to evaluate if a product was delivered in time or not and the genuine product trajectories were inferred using a frequent pattern mining algorithm. Another example of spatial-temporal analysis of data is in the field of crime investigation. Esquivel et al. [30] employed an artificial neural network to build models in order to predict future crimes based on past patterns [31] through the development of a genetic-fuzzy system which encompasses spatial-temporal patterns for predicting future crimes.

### 3.2. Examples of Spatial-Temporal Data Analysis Applications in the Food Traceability Domain

In the framework of food quality control, spatial-temporal data analysis could represent an important advance for the assurance of product traceability.

In this sense, the analysis of spatial-temporal data could help to extend the use, for example, of QR codes or RFID tags for food fraud detection since these identifiers themselves are not that important; it is the information that is retrieved from them about a certain product batch, and the time and location of the scan. When an identifier is scanned, this is registered, together with the location and moment the code is scanned (if not restricted by the user). In a real-life situation, the scanning logs would reveal if the products sent to location A were indeed scanned in the location and/or surroundings in a given period in which it is expected to be sold (before its sell-by date). It would also show if the number of scans reflect what is typically expected (e.g., 10–20% of all products are scanned). Combining location and the number of scans in a given time period would generate a certain pattern. Hence, when looking at the spatial dimension (where products are scanned) and the temporal patterns (when products are scanned) it would be possible to establish if everything is normal or not. These patterns can be described and generated with algorithms to determine both situations.

For example, this approach could detect copied or reused product packaging, since the packaging would likely still contain the identifiers, and when scanned, the locations and time (dates) will be unusual. This may be after the sell-by dates of the products or scanned in areas where the products were never sent to, or one may see an unusual high number of identifiers being scanned (>>10–20%, for instance, that was expected to be scanned). This will flag anomalies and be an indication (early warning) of counterfeited products.

In this way, it will allow the detection of reiterative, yet novel and useful behaviour, through the evaluation of patterns to be able to assess whether the behaviour of the product within the distribution chain is normal or not. A pattern can be defined as a constant and recurrent characteristic or feature that helps to identify a phenomenon or problem and serves as an indicator or model to predict its future behaviour. It should be noted that the analysis of this type of data requires the application of a multivariate approach using machine learning methods (ML), which are a combination of many different methods, among which are two central concepts, artificial neural networks (ANN) and deep learning (DL).

**Figure 1:**
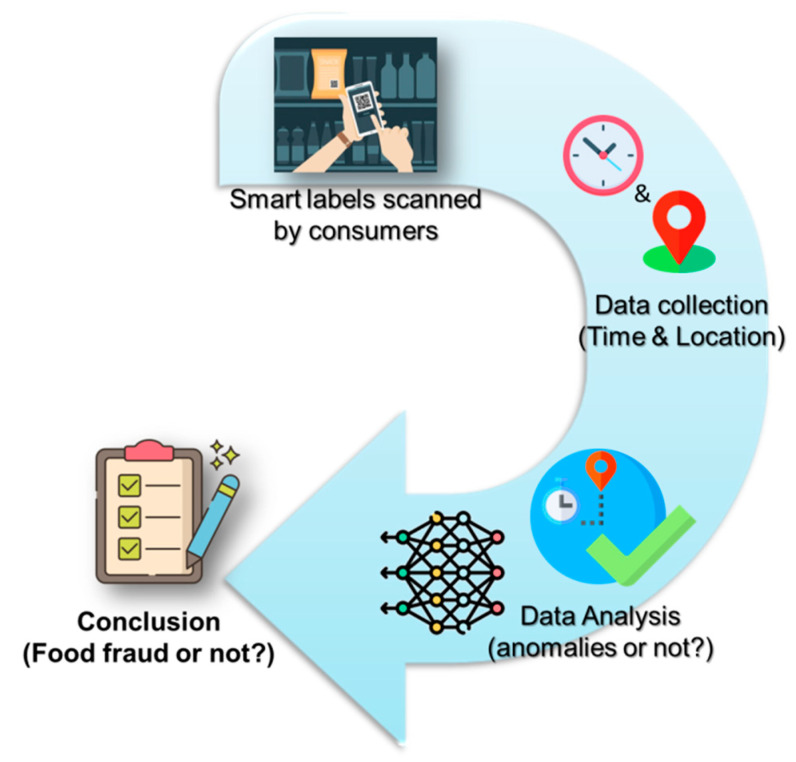
Outline of the use of spatial-temporal analysis of data collected from consumers who scan QR or other smart labels.

The spatial-temporal data analysis could be applied to scans taken along the chain in traceability systems. However, the approach could also focus on consumers who scan QR codes in retail outlets. Figure 1 depicts the general workflow of the latter variant.

The key to making the most of this multivariate approach is to know the type of data being collected, how to structure and organise it, and which type of multivariate technique is most appropriate or has the greatest potential. For example, ML can help detect possible anomalies since from a properly trained and validated multivariate model it would be possible to assess whether a new spatial-temporal data vector of a food behaves according to the built model or not. 

It is in this context which some authors have proposed the use of spatial-temporal data analysis to control the traceability of some food products such as wine, Ziziphus jujuba (Chinese red date) and meat in a digital way [32,33,34]. The most common frauds in the wine sectors can be categorised as ′ counterfeiting′, ′packaging recycling′, and even ′product overrun′. In this context, Popivić et al. [32] proposed the use of a mobile app in which every time users scan a QR code (which uniquely identifies a product), they provide an update on the status and location of that bottle. They carried out a pilot study with some companies in the sector that export their wine bottles to different countries. To ensure the authenticity of the wine bottles, the authors designed a smart label for each bottle, which consisted of a QR code, and a letter stamped on the bottle using invisible photochromatic functional ink. The mandatory requirement to receive authenticity confirmation is to successfully match the QR code—letter code. Therefore, each bottle was individually tracked and traced throughout the supply chain and the information updates were used to identify whether there was a potential counterfeit issue with a wine bottle.

Sun et al. [33] designed an anti-counterfeit system for identifying the origin of Chinese red dates based on GPS coordinates and encrypted Chinese-sensible codes per product. They selected several product characteristics (i.e., product weight, GPS coordinates, origin, and product code) and subsequently generated dedicated codes that were stamped on the products. In this way, when the products moved from one place to another, the QR codes were read, and the locations were evaluated using the GPS coordinates with the goal of detecting any anomaly throughout the supply chain. The results showed that 6310 of the product labels that were generated were correctly identified and allowed for the tracing of the origin of the products to the end consumer, resulting in a recognition rate of approximately 98%. The other 2% of product labels were not identified, and according to the authors, this was caused by the deterioration of the smart codes.

Similarly, Ren et al. [34] employed RFID technology to assess the traceability of meat products through spatial-temporal data analysis. Specifically, they developed software that tracked the information of the meat with the RFID tag in such a way that it could be known in which shop the meat was purchased. Finally, they compared the application of this promising approach with the traditional traceability system based on barcode reading, demonstrating the potential of this system, since it has faster data acquisition, a higher reorganisation rate and involves a more automated process.

### 3.3. Adaptation of Spatial-Temporal Data Analysis in Food Traceability Systems for Early Warning of Food Authenticity Infringements

As far as the adaptation of this approach is concerned, it should be noted at this point that the volume of available spatial-temporal information will be largely influenced by consumer behaviour, since this is spontaneous and hardly controllable. However, it would be possible to assume some hypothetical scenarios in which the scanning of a smart tag could be concentrated. For example, the scanning locations may be more concentrated at the locations of the retailer, the scanning time is more likely to appear in the peak shopping time period of retailers, and the daily scanning number of a certain area may be within a reasonable range. Thus, out of this hypothetical scenario it could be assumed that if there are anomalies in the spatial-temporal patterns it could be due to food fraud, such as:

Counterfeiting: the smart tag (QR code, RFID tag etc.) is copied and pasted on many counterfeit foods illegally, and the smart tag will be scanned many times in many unexpected areas.

Theft and diversion may lead to the authentic food being sold by the expected retailers, and the locations of scanning the smart tag do not occur in the retailers.

Packaging recycling: the smart tag is exposed to consumers again, and consumers may scan the QR code at an unexpected time and location.

In this regard, some publications have suggested their use for following products en route and in some cases in detecting one type of food fraud, such as counterfeiting, e.g., for the fraud detection of champagne [35]. However, there has been limited use of the spatial-temporal information for the purpose of crime detection, with only a few studies demonstrating its potential (Section 3.2). It is probable that the use of spatial-temporal data analysis to detect food fraud has not expanded due to several challenges. Firstly, most food companies have been not fully aware of the potential of the spatial-temporal information about consumer scanning of the smart labels to detect food fraud. At present, there are few articles or reports illustrating the feasibility of the spatial-temporal information in fraud detection. Secondly, a system for capturing and storing the spatial-temporal data needs to be generated. As smart tags are usually printed on food packaging and scanned by different consumers at different times and places, it is necessary to have software available that is capable of processing this data online to provide a quick response on the status of the product for this complex situation [36]. Lastly, there is always a cost/benefit balance aspect, and there is some question as to whether it is worth it to invest in these systems and whether they will they result in sufficient benefits.

## 4. Future Implications and Perspectives

Currently, different information systems exist, such as smart tags and QR codes, which aim to provide consumers with more information and to generate more transparency regarding the food consumed, or to ensure that the data is not altered once it is generated (as is the case of Blockchain technology). However, the analysis of spatial-temporal data together with the application of artificial intelligence could lead to further advancements in fighting food fraud, as it could be used as an early warning food fraud tool given that the position, time and/or additional information of the product would be obtained when its identification code is scanned. Conversely, it should be noted that to implement such a tool, it is necessary to face several challenges: generating a system for capturing and storing the time-space data and producing software capable of quickly processing the data in real time to provide a rapid response on the status of the product, while another challenge involves the availability, security and ownership of information throughout the supply chain.

## Data Availability

The data presented in this study are available on request from the corresponding author.
